# Supplementary material for: How migration shapes modern contraceptive use among urban young women: Evidence from six African countries
Source: PLoS One. 2024 Jul 23;19(7):e0307141. doi: 10.1371/journal.pone.0307141 (PMC11265688; doi:10.1371/journal.pone.0307141)
Supplement: S1 Appendix — (DOCX) [file pone.0307141.s002.docx]

**Appendix 1a: Pairwise comparisons for modern method use comparing each sequence for MCP***

|  | None | Mig-Mar-Birth | Mig-Birth-Mar | Mig-Mar | Mig-Birth | Mig | Mar-Mig-Birth | Mar-Birth-Mig | Mar-Mig | Mar-Birth | Mar | Birth-Mig-Mar | Birth-Mar-Mig | Birth-Mar | Birth-Mig |
| --- | --- | --- | --- | --- | --- | --- | --- | --- | --- | --- | --- | --- | --- | --- | --- |
| Mig-Mar-Birth | 0.09 |  |  |  |  |  |  |  |  |  |  |  |  |  |  |
| Mig-Birth-Mar | 0.18 | 0.08 |  |  |  |  |  |  |  |  |  |  |  |  |  |
| Mig-Mar | -0.19 | -0.28 | -0.36 |  |  |  |  |  |  |  |  |  |  |  |  |
| Mig-Birth | 0.04 | -0.05 | -0.13 | 0.23 |  |  |  |  |  |  |  |  |  |  |  |
| Mig | 0.03 | -0.07 | -0.15 | 0.21 | -0.02 |  |  |  |  |  |  |  |  |  |  |
| Mar-Mig-Birth | 0.11 | 0.02 | -0.06 | 0.3 | 0.07 | 0.08 |  |  |  |  |  |  |  |  |  |
| Mar-Birth-Mig | 0.08 | -0.01 | -0.1 | 0.26 | 0.04 | 0.05 | -0.03 |  |  |  |  |  |  |  |  |
| Mar-Mig | -0.24 | -0.33 | -0.41 | -0.05 | -0.28 | -0.27 | -0.35 | -0.32 |  |  |  |  |  |  |  |
| Mar-Birth | 0 | -0.09 | -0.18 | 0.18 | -0.04 | -0.03 | -0.11 | -0.08 | 0.24 |  |  |  |  |  |  |
| Mar | -0.19 | -0.28 | -0.36 | 0 | -0.23 | 0.21 | -0.29 | -0.26 | 0.05 | -0.18 |  |  |  |  |  |
| Birth-Mig-Mar | 0.11 | 0.01 | -0.07 | 0.29 | 0.06 | 0.08 | 0 | 0.03 | 0.34 | 0.11 | 0.29 |  |  |  |  |
| Birth-Mar-Mig | 0.16 | 0.07 | -0.02 | 0.34 | 0.12 | 0.13 | 0.05 | 0.08 | 0.4 | 0.16 | 0.34 | 0.05 |  |  |  |
| Birth-Mar | 0.09 | 0.01 | -0.08 | 0.28 | 0.06 | 0.07 | -0.01 | 0.02 | 0.34 | 0.1 | 0.28 | -0.01 | -0.06 |  |  |
| Birth-Mig | 0.12 | 0.02 | -0.06 | 0.3 | 0.07 | 0.09 | 0.01 | 0.04 | 0.36 | 0.12 | 0.3 | 0.01 | -0.04 | 0.02 |  |
| Birth | 0.04 | -0.05 | -0.14 | 0.22 | -0.01 | 0.01 | -0.07 | -0.04 | 0.28 | 0.04 | 0.22 | -0.07 | -0.1 | -0.06 | -0.08 |

*Red values are statistically significant (p<0.05) and negatively correlated, while green values are statistically significant and positively correlated

**Appendix 1b: Pairwise comparisons for modern method use comparing each sequence for recent HF visit***

|  | None | Mig-Mar-Birth | Mig-Birth-Mar | Mig-Mar | Mig-Birth | Mig | Mar-Mig-Birth | Mar-Birth-Mig | Mar-Mig | Mar-Birth | Mar | Birth-Mig-Mar | Birth-Mar-Mig | Birth-Mar | Birth-Mig |
| --- | --- | --- | --- | --- | --- | --- | --- | --- | --- | --- | --- | --- | --- | --- | --- |
| Mig-Mar-Birth | 0.43 |  |  |  |  |  |  |  |  |  |  |  |  |  |  |
| Mig-Birth-Mar | 0.35 | -0.08 |  |  |  |  |  |  |  |  |  |  |  |  |  |
| Mig-Mar | 0.18 | -0.25 | -0.17 |  |  |  |  |  |  |  |  |  |  |  |  |
| Mig-Birth | 0.29 | -0.14 | -0.05 | 0.11 |  |  |  |  |  |  |  |  |  |  |  |
| Mig | 0.04 | 0.4 | -0.31 | -0.15 | -0.26 |  |  |  |  |  |  |  |  |  |  |
| Mar-Mig-Birth | 0.41 | -0.02 | 0.06 | 0.23 | 0.11 | 0.37 |  |  |  |  |  |  |  |  |  |
| Mar-Birth-Mig | 0.31 | -0.12 | -0.03 | 0.13 | 0.02 | 0.28 | -0.09 |  |  |  |  |  |  |  |  |
| Mar-Mig | 0.1 | -0.34 | -0.25 | -0.09 | -0.2 | 0.06 | -0.31 | -0.21 |  |  |  |  |  |  |  |
| Mar-Birth | 0.32 | -0.11 | -0.03 | 0.14 | 0.04 | 0.29 | -0.09 | 0.01 | 0.22 |  |  |  |  |  |  |
| Mar | 0.09 | -0.34 | -0.26 | -0.09 | -0.2 | 0.05 | -0.32 | -0.23 | -0.01 | -0.23 |  |  |  |  |  |
| Birth-Mig-Mar | 0.32 | -0.12 | -0.04 | 0.13 | 0.02 | 0.28 | -0.1 | 0 | 0.21 | -0.01 | 0.22 |  |  |  |  |
| Birth-Mar-Mig | 0.31 | -0.14 | -0.05 | 0.11 | 0 | 0.26 | -0.11 | -0.02 | 0.2 | -0.03 | 0.21 | -0.02 |  |  |  |
| Birth-Mar | 0.32 | -0.12 | -0.03 | 0.14 | 0.02 | 0.28 | -0.09 | 0 | 0.22 | -0.01 | 0.23 | 0.01 | 0.02 |  |  |
| Birth-Mig | 0.15 | -0.31 | -0.2 | -0.01 | -0.13 | 0.13 | -0.25 | -0.17 | 0.06 | -0.16 | 0.07 | -0.15 | -0.14 | -0.16 |  |
| Birth | 0.25 | -0.19 | -0.1 | 0.07 | -0.04 | 0.21 | -0.16 | -0.07 | 0.15 | -0.07 | 0.16 | -0.06 | -0.05 | -0.07 | 0.09 |

*Red values are statistically significant (p<0.05) and negatively correlated, while green values are statistically significant and positively correlated
