# Supplementary material for: How migration shapes modern contraceptive use among urban young women: Evidence from six African countries
Source: PLoS One. 2024 Jul 23;19(7):e0307141. doi: 10.1371/journal.pone.0307141 (PMC11265688; doi:10.1371/journal.pone.0307141)
Supplement: Attachment — Submitted filename: PLOS ONE REVIEWER FEEDBACK.docx [file pone.0307141.s003.docx]

**Title**

The authors have addressed an important area of population health that link migration to facility visits and contraceptive among use adolescent girls and young women. I have generally enjoyed reading the manuscript but there are issues that must be addressed before the paper may be considered for publication.

**Abstract:**

1.There is minor grammatical error in the abstract line 31

2. May the authors include key results from their logistic regression model (Odds or Odds ratios and their corresponding 95% confidence intervals) in their result section of their abstract.

**Background**

The authors have presented important information in the background. However, they have not indicated the proportion of migrants that are young aged 15 to 24. Demographic and Health Surveys have information on these estimates for both. The authors alluded to the scarcity of studies on migration and family planning use, I agree with the authors but there are studies they could have referenced including their quantitative findings e.g. contraceptive prevalence rate among young women, odd ratio of contraceptive use comparing migrants to non-migrants.

**Methods**

This study used data collected in 6 African countries. Observations within countries could be correlated; this should have been investigated to assess evidence or significant correlation. The authors needed to apply multilevel modeling approach or models that can adjust for clustering of observations within countries other than using naïve logistic regression model. The clustering could even apply at countries level if there were clusters within countries. Use of naïve logistic regression violates the independence of observations assumption of the generalized linear models to which logistic is part of. These need to be addressed.

**Discussion**

The discussion is well written. The authors mentioned in the discussion that there were wide variations in the exposure (migration) and outcomes, this is consistent with clustering as suggested in the methods section. The authors will need to rework results based on my suggestion in methods and update both results and methods.
